# *Lacticaseibacillus rhamnosus* HN001 alters the microbiota composition in the cecum but not the feces in a piglet model

**DOI:** 10.3389/fnut.2022.1002369

**Published:** 2022-10-28

**Authors:** Wayne Young, Paul Maclean, Kelly Dunstan, Leigh Ryan, Jason Peters, Kelly Armstrong, Rachel Anderson, Hilary Dewhurst, Melanie van Gendt, Ryan N. Dilger, James Dekker, Neill Haggarty, Nicole Roy

**Affiliations:** ^1^AgResearch, Te Ohu Rangahau Kai, Palmerston North, New Zealand; ^2^Riddet Institute, Massey University, Palmerston North, New Zealand; ^3^Department of Animal Sciences, University of Illinois, Urbana, IL, United States; ^4^Fonterra Research and Development Centre, Palmerston North, New Zealand

**Keywords:** probiotic, *Lacticaseibacillus rhamnosus*, HN001, piglet, microbiome, gut

## Abstract

The probiotic *Lacticaseibacillus rhamnosus* strain HN001 has been shown to have several beneficial health effects for both pediatric and maternal groups, including reduced risk of eczema in infants and gestational diabetes and postnatal depression in mothers. While *L. rhamnosus* HN001 appears to modify immune and gut barrier biomarkers, its mode of action remains to be fully elucidated. To gain insights into the role of HN001 on the infant microbiome, the impacts of *L. rhamnosus* HN001 supplementation was studied in 10-day old male piglets that were fed either infant formula, or infant formula with *L. rhamnosus* HN001 at a low (1.3 × 10^5^ CFU/ml) or high dose (7.9 × 10^6^ CFU/ml) daily for 24 days. The cecal and fecal microbial communities were assessed by shotgun metagenome sequencing and host gene expression in the cecum and colon tissue was assessed by RNA-seq. Piglet fecal samples showed only modest differences between controls and those receiving dietary *L. rhamnosus* HN001. However, striking differences between the three groups were observed for cecal samples. While total lactobacilli were significantly increased only in the high dose *L. rhamnosus* HN001 group, both high and low dose groups showed an up to twofold reduction across the *Firmicutes* phylum and up to fourfold increase in *Prevotella* compared to controls. *Methanobrevibacter* was also decreased in HN001 fed piglets. Microbial genes involved in carbohydrate and vitamin metabolism were among those that differed in relative abundance between those with and without *L. rhamnosus* HN001. Changes in the cecal microbiome were accompanied by increased expression of tight junction pathway genes and decreased autophagy pathway genes in the cecal tissue of piglets fed the higher dose of *L. rhamnosus* HN001. Our findings showed supplementation with *L. rhamnosus* HN001 caused substantial changes in the cecal microbiome with likely consequences for key microbial metabolic pathways. Host gene expression changes in the cecum support previous research showing *L. rhamnosus* HN001 beneficially impacts intestinal barrier function. We show that fecal samples may not adequately reflect microbiome composition higher in the gastrointestinal tract, with the implication that effects of probiotic consumption may be missed by examining only the fecal microbiome.

## Introduction

Probiotics are defined as living microorganisms that confer health benefits on the host when consumed in adequate amounts (1). They are typically lactic acid bacteria belonging to the *Lactobacillus* or *Bifidobacterium* genera. Due to their demonstrated health benefits, they are commonly added to infant formula (2).

Several beneficial health effects have been reported for the probiotic *Lacticaseibacillus rhamnosus* (formerly *Lactobacillus rhamnosus*) strain HN001 for both pediatric and maternal groups, including reduced risk of eczema in infants (3) and reduced risk of gestational diabetes and postnatal depression in mothers (4). Effects may also be long lasting, with evidence showing early childhood supplementation with *L. rhamnosus* HN001 can lead to reduced incidence of eczema at 11 years of age (5). While *L. rhamnosus* HN001 appears to modify immune and gut barrier biomarkers (6), its mode of action remains to be fully elucidated. Consumption of *L. rhamnosus* HN001 does not tend to result in colonization (7) and a recent study of infant microbiomes from fecal samples collected as part of an anti-eczema study showed few significant changes in the microbiome, both in terms of taxa and metabolic pathways (8).

The effects of probiotics are likely to occur through several mechanisms, individually or in combination, depending on the specific strain of bacterium. One possible mechanism is through the improvement of intestinal barrier integrity (9) to reduce the translocation of antigens across the gut epithelium. Probiotics may also modulate the host immune system *via* specific receptors that sense microbial metabolites such as short chain fatty acids (SCFAs) and polysaccharides (10–12). A further putative mechanism is the influence of the probiotic on the microbiota present in the gut. However, the extent to which probiotics are able to modulate the resident microbiota is unclear, with some studies showing little effect (13) and others showing that effects are dependent on the starting microbiome (14). A contributing factor to the variable demonstrated impacts of probiotics on the gut microbiome may be that most studies measure changes in the fecal microbiome, which may not fully reflect the situation in the gastrointestinal tract itself (15).

During early life, a probiotic added to infant formula may shape the large intestinal environment into one that promotes a more beneficial microbiota, and thus enhance its development and maturation. In this study, we aimed to better understand the impact of *L. rhamnosus* HN001 in early life on the gastrointestinal microbiome using piglets fed an infant formula supplemented with two different doses of *L. rhamnosus* HN001. Compared with rodents, the neonatal pig shares greater similarities with neonatal humans in terms of anatomical and physiological features (16, 17). The cecal and fecal microbial communities were compared using shotgun metagenome sequencing. Associated changes in the cecal and colonic tissue gene expression profiles were compared using RNA-seq.

## Materials and methods

### Animals

The study was carried out in strict accordance with the NZ Animal Welfare Act 1999, and was approved by the AgResearch Limited (Grasslands) Animal Ethics Committee (Ethics Approval No.: 13982).

Twenty-four male large white cross 10-day old piglets were obtained from a commercial farm in the Manawatu-Wanganui region of New Zealand. All piglets were housed in custom cages constructed to allow animals to see, hear, and smell adjacent piglets while minimizing physical contact (18). On arrival at the animal facility (day 1), the piglets were pair-housed for two nights. The piglets were exclusively fed reconstituted infant formula [control formula; Fonterra Nutritional Base (protein 13.63%, fat 26.2%, ash 2.8%, carbohydrate 55.07%) + DCL100 at 5 g/L of formula] 2 h post-arrival and every 4 h thereafter. From day 3 to day 24 the piglets were individually housed. During this period, the piglets were let out into a shared pen and allowed to physically interact for an hour of social time each day.

*Lactocaseibacillus rhamnosus* strain HN001 (formerly known as *Lactobacillus rhamnosus* HN001, also known as LactoB HN001™ or DR20™) was supplied by Fonterra (Palmerston North, New Zealand).

From day 3 to day 24, the piglets were assigned to one of three treatment groups; 8 receiving control formula; 8 receiving control formula supplemented with 1.3 × 10^5^ CFU/ml of *L. rhamnosus* HN001 (HN001 Low); and 8 receiving control formula supplemented with 7.9 × 10^6^ CFU/ml of *L. rhamnosus* HN001 (HN001 High). The doses used were within the range of commonly used amounts for infant formulae (19). All formulas were dispensed using an automated system programmed to offer the formula every 2 h (starting at 2 L/day on day 3, increasing to 5.8 L/day by day 24) with automatic measurement of refusals. The piglet caging and automated feeding systems were designed and manufactured by ShapeMaster (Ogden, IL, USA).

Fecal samples were collected each day and stored at −80°C for later analyses. At the end of the study, the piglets were euthanized by sedation using a cocktail of tiletamine hydrochloride, zolazepam hydrochloride, xylazine, and ketamine hydrochloride (final solution 50 mg/ml of each drug, administered at a dose rate of 0.3 mL of the mixed solution/10 kg body weight), followed by intracardiac puncture with sodium pentobarbitone (300 mg/ml; 0.27 ml/kg body weight). Cecal contents were collected and stored at −80°C for microbiome analyses. Tissue samples from the cecum and ascending colon were collected into RNAlater (Qiagen, Hilden, Germany) and stored at −80°C for gene expression analysis.

### Microbiome

Metagenomic DNA was extracted from cecal contents and fecal samples from the final day using Macherey Nagel NucleoSpin Soil kits (Düren, Germany) following the manufacturer’s instructions, with the addition of bead beating on a BioSpec Mini-Beadbeater 96 (Bartlesville, OK, USA) set to 4 min at 40 oscillations/s.

A total amount of 1 μg of metagenomic DNA per sample was used as input material for the DNA sample preparations. Sequencing libraries were generated using NEBNext^®^ Ultra™ DNA Library Prep Kit for Illumina (New England Biolab, Ipswich, MA, USA) following the manufacturer’s recommendations and index codes were added to attribute sequences to each sample. Briefly, the DNA sample was fragmented by sonication to a size of 300 bp, then DNA fragments were end-polished, A-tailed, and ligated with the full-length adaptor for Illumina sequencing with further PCR amplification. The PCR products were purified (AMPure XP system) and libraries were analyzed for size distribution by the Agilent 2100 Bioanalyzer and quantified using real-time PCR. Clustering of index-coded samples was performed on a cBot Cluster Generation System according to the manufacturer’s instructions. After cluster generation, the library preparations were sequenced on an Illumina HiSeq X platform and 150-bp paired-end reads were generated.

The software Trimmomatic v.0.36 (20) was used for removal of adapters, low quality (Phred scores < 30), and short (<50 bp) sequencing reads. Read pairs were joined using PEAR version 0.9.6 (21) with default settings. Read pairs that did not join were concatenated with a spacer consisting of a string of N’s using the “fuse” function from the BBMAP package version 38.22-0 (22). Evaluation and detection of host reads were done using the bbduk.sh function from the BBMAP package version 38.22-0 (22), a k-mer based filter, with the pig genome (Sscrofa 11.1 release 96) as reference. The “blastx” function of DIAMOND version 0.9.22 (23) was used to map the reads against the “nr” NCBI database. MEGAN6 Ultimate Edition (24) was used to assign putative functions to the DIAMOND alignment files against the SEED Subsystems database (25).

Comparisons of overall community compositions were performed using permutational multivariate analysis of variance (PERMANOVA) of distance matrices, implemented through the adonis function from the vegan package (26) for R. Differences in the relative abundances of individual taxa and gene functions were analyzed using ANCOM-BC (27) package in R, with *Q*-values < 0.05 considered significant. ANCOM-BC is a method for detecting differences in abundance while accounting for the compositional nature of microbiome datasets.

### Piglet gut tissue transcriptome

Gene expression profiles from cecal and colon tissue samples were analyzed by RNA-seq. Total RNA was extracted from samples using RNeasy Mini Kits (Qiagen, Germantown, MD, USA). Total RNA quality and quantity were determined using an Agilent 2100 Bioanalyzer Instrument (Agilent, Santa Clara, CA, USA) and Nanodrop (Thermo Fisher Scientific, Waltham, MA, USA), and sample quality was also assessed using agarose gel electrophoresis. Samples that passed the RNA integrity number (RIN) threshold of 6.5 were submitted for sequencing. Strand-specific cDNA libraries were prepared using NEBNext^®^ Ultra Directional RNA Library Prep Kit for Illumina^®^ (New England Biolab, Ipswich, MA, USA) according to the manufacturer’s guidelines. Libraries were size selected for 250–300 bp fragments and sequenced using the Novaseq 6000 platform (Illumina) to produce 150 bp paired-end sequences. The reads were quality trimmed using Trimmomatic 0.36 (20) in paired-end mode using the following parameters; LEADING:3 TRAILING:3 SLIDINGWINDOW:4:15 MINLEN:36. Read pairs that passed quality trimming were mapped against the genome (Sscrofa 11.1 release 96) using STAR (28). Uniquely mapped read pairs were summed for each gene and analyzed using the EdgeR package (29) in R. The resulting counts were analyzed using a likelihood ratio generalized linear model, with genes that had > 1.5-fold difference (i.e., log fold change > | 0.58|) and FDR < 0.05 considered differentially expressed. Gene expression profiles were also analyzed by gene set enrichment analysis (GSEA) using the mroast function from limma (30) and KEGG pathways (31, 32) as gene sets. GSEA involves the analysis of the collective expression of groups of genes treated as a unit rather than as individual genes.

## Results

There were no significant differences in body weight between the groups at the start of the study (*P* = 0.96) or at the conclusion of the study (*P* = 0.94). Similarly, no differences in the percentage weight gain were observed (*P* = 0.95). The amount of infant formula consumed over the course of the study did not differ between groups (*P* = 0.31) and no differences in animal health or adverse effects were apparent.

Sequence reads are available from the NCBI Sequence Read Archive (SRA), accession PRJNA823879.

### Microbiome

Following quality trimming the median number of paired-end reads per sample was 10.7 M, with a standard deviation, minimum, and maximum number of reads of 1.8, 6.8, and 15.6 M, respectively.

#### *Lacticaseibacillus rhamnosus* relative abundances

**FIGURE 1:**
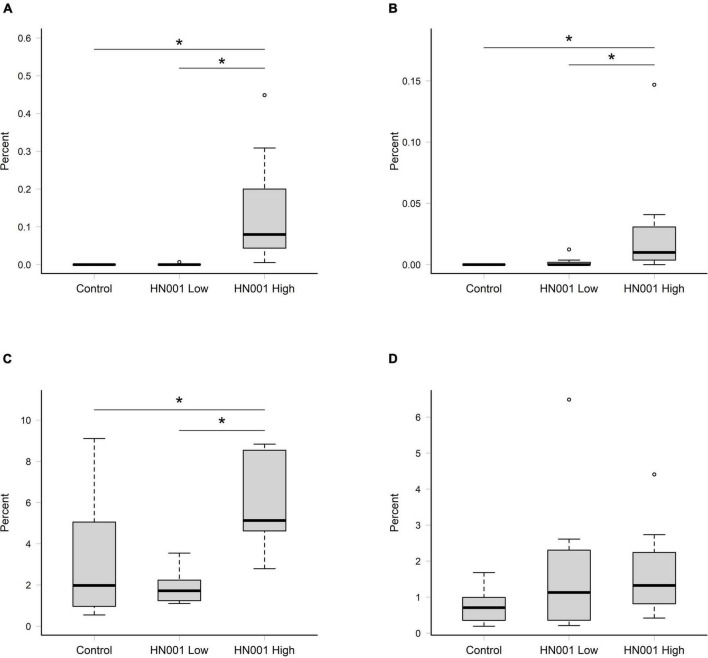
Relative abundances of reads assigned to *Lacticaseibacillus rhamnosus* in **(A)** cecal and **(B)** fecal samples, and the collective relative abundances of reads assigned to *L. rhamnosus* and uncultured and unclassified *Lactobacillus* in in **(C)** cecal and **(D)** fecal samples. Significant differences indicated by asterisk (permutation ANOVA *P* < 0.01). Boxplots indicate median (middle line), first and third quartile (boundaries of box), 1.5 times the interquartile range (whiskers), and outliers (circles).

The taxonomic data summarized at the species level from the MEGAN analysis of NCBI nr hits, showed supplementation with *L. rhamnosus* HN001 resulted in a higher relative abundance of *L. rhamnosus* in the cecal and fecal community (*P* < 0.001 and *P* = 0.007, respectively) at the higher dose, compared to the Control and HN001 Low groups (Figures 1A,B). Because of the uncertainty with classification to the species level (33, 34), we also considered the relative abundance of taxa classified as uncultured and unclassified *Lactobacillus* (which in this instance includes *Lacticaseibacillus* and other former members of the recently reclassified *Lactobacillus* genus). When these were combined with sequences identified as *L. rhamnosus*, we saw a similar pattern in the cecum where the relative abundance of these combined sequences was higher in the HN001 High group (*P* = 0.018; Figure 1C). However, no differences were observed in the fecal community (*P* = 0.232; Figure 1D).

#### Cecal microbiome

**FIGURE 2:**
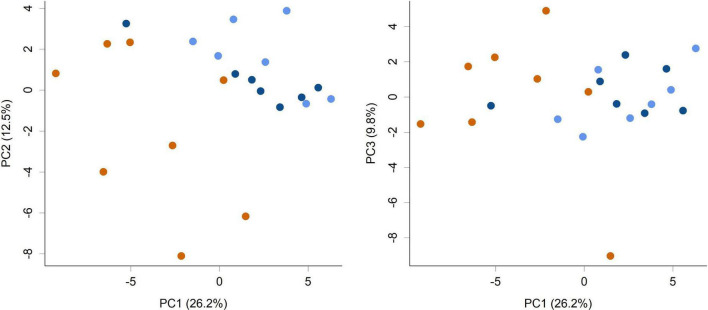
Principal component analysis (PCA) scores plot of the piglet cecal microbiome community composition at the genus level (PC1 vs. PC2 and PC1 vs. PC3 shown). Colors indicate groups; Control (orange), HN001 Low (light blue), HN001 High (dark blue). Permutation MANOVA *P* = 0.001 indicates groups had significantly different compositions. Pairwise permutation MANOVAs showed the HN001 Low and HN001 High were not different to each other (*P* = 0.283), whereas Controls differed from both HN001 High (*P* = 0.012) and HN001 Low (*P* = 0.002).

The supplementation of infant formula with *L. rhamnosus* HN001 also led to dramatic changes in the overall cecal microbiome composition (Figure 2, PERMANOVA *P* = 0.001, Table 1). This included a large increase in Gram-negative phyla. Piglets in both HN001 High and HN001 Low groups had increased relative abundance of *Bacteroidetes* compared to the Controls (global *Q* < 0.001). Similarly, both doses of HN001 increased relative abundances of *Proteobacteria* (global *Q* = 0.03). At the genus level *Prevotella* were relatively more abundant in piglets fed either dose of *L. rhamnosus* HN001 compared to Control (*Q* < 0.05). The change in *Prevotella* was particularly notable as they underwent a major fold change while also comprising a substantial proportion of the community (Control 8.9% ± 1.7; HN001 Low 23.9% ± 3.1; HN001 High 17.6% ± 3.3; mean% ± SEM).

**TABLE 1:** Microbial taxa at the genus level and higher with > 0.1% mean relative abundances in the cecum with significant differences (global ANCOM-BC *Q* < 0.05) between piglets fed infant formula supplemented with *L. rhamnosus* HN001 and control formula.

Phylum	Family	Genus	Control	HN001 Low	HN001 High	*P*	*Q*	*W*
*Euryarchaeota*	*Methanobacteriaceae*	*Methanobrevibacter*	0.29 ± 0.06	0.11 ± 0.04*	0.09 ± 0.04*	0.004	0.031	12.091
*Bacteroidetes*			32.92 ± 3.23	46.58 ± 4.05*	38.5 ± 3*	<0.001	<0.001	39.069
*Bacteroidetes*	*Prevotellaceae*		10.74 ± 2.06	27.03 ± 3.16*	19.86 ± 3.57*	<0.001	0.002	21.539
*Bacteroidetes*	*Prevotellaceae*	*Prevotella*	8.85 ± 1.69	23.85 ± 3.05*	17.58 ± 3.25*	<0.001	0.002	18.956
*Bacteroidetes*	*Prevotellaceae*	uncl. *Prevotellaceae*	1.79 ± 0.52	3.03 ± 0.45*	2.2 ± 0.4	0.003	0.024	13.046
*Firmicutes*			33.86 ± 3.43	18.73 ± 2.40*	21.87 ± 2.32*	0.029	0.053	2.970
*Firmicutes*	*Lactobacillaceae*	*Lactobacillus*	1.42 ± 0.52	0.74 ± 0.12	3.61 ± 0.81*	<0.001	0.005	16.832
*Firmicutes*	uncl. *Clostridiales*		8.36 ± 1.13	3.07 ± 0.61*	4 ± 0.48*	0.006	0.034	11.723
*Firmicutes*	*Clostridiales*	uncl. *Clostridia*	0.37 ± 0.05	0.12 ± 0.03	0.15 ± 0.03	0.001	0.005	16.524
*Proteobacteria*			14.58 ± 2.89	22.88 ± 4.04*	26.61 ± 5.06*	0.001	0.003	15.38
*Proteobacteria*	*Enterobacteriaceae*	*Citrobacter*	0.07 ± 0.03	0.19 ± 0.14*	0.49 ± 0.16*	<0.001	0.005	16.74

Mean percent ± standard error of means. *Indicates significantly different to Control (ANCOM-BC *Q* < 0.05).

The increase in Gram-negative bacteria was accompanied by a concomitant decrease in Gram-positive bacteria including the *Firmicutes* (global *Q* = 0.05) and *Actinobacteria*, although the latter was not considered a significant difference (*Q* = 0.11). *Methanobrevibacter* was also lower in piglets receiving HN001 (*Q* = 0.004; Control 0.29 ± 0.06; HN001 Low 0.11 ± 0.04; HN001 High 0.09 ± 0.04; mean% ± SEM). An exception to the overall decrease in Gram-positive bacteria was *Lactobacillus*, which was significantly higher in piglets fed the high dose of HN001 compared to the Control group (*Q* < 0.01).

Changes to the cecal microbiome were also apparent in the differences in the relative abundances of genes related to a wide range of metabolic processes and pathways. Analysis of genes mapped to SEED Subsystems level 2 functions showed 158 out of 597 functions had significantly different abundances (global ANCOM-BC *Q* < 0.05). The 50 most relatively abundant significantly different level 2 functions are shown in Table 2. These included genes involved in carbohydrate metabolism; genes annotated to the SEED function *Lactate utilization* were more relative abundant in the HN001 Low and HN001 High group compared to Controls (*Q* < 0.001), while genes categorized to *Pyruvate:ferredoxin oxidoreductase* and *Methanogenesis* were lower in relative abundance for both HN001 groups compared to Controls (*Q* < 0.001). Other SEED categories related to methanogens and methane metabolism also differed in relative abundance; *Carbon monoxide induced hydrogenase, H_2_:CoM-S-S-HTP oxidoreductase*, and *Aromatic amino acid interconversions with aryl acids* were also lowered in both HN001 groups compared to Controls (*Q* < 0.05), while genes assigned to *Formate dehydrogenase* function were increased in piglets receiving HN001 (*Q* = 0.009).

**TABLE 2:** SEED level 1 functions and top 50 most abundant microbial SEED level 2 functions with significantly different (global ANCOM-BC *Q* < 0.05) mean relative abundances in the cecum of piglets fed infant formula supplemented with *L. rhamnosus* HN001 and control formula.

SEED function (level 1; level 2)	Control	HN001 low	HN001 high	*Q*	*W*
Amino acids and derivatives; arginine and ornithine degradation	0.258 ± 0.014	0.299 ± 0.018*	0.312 ± 0.029*	0.0029	17.8576
Amino acids and derivatives; aromatic amino acid degradation	0.028 ± 0.007	0.046 ± 0.008	0.054 ± 0.013*	0.0466	10.2052
Amino acids and derivatives; aromatic amino acid interconversions with aryl acids	0.06 ± 0.007	0.029 ± 0.004*	0.031 ± 0.006*	0.0427	10.4808
Amino acids and derivatives; threonine anaerobic catabolism gene cluster	0.04 ± 0.002	0.05 ± 0.003*	0.05 ± 0.003*	0.0002	25.0241
**Carbohydrates**	**5.635 ± 0.177**	**6.146 ± 0.221** *	**6.387 ± 0.326** *	**0.0011**	**19.2067**
Carbohydrates; acinetobacter tca	0.187 ± 0.015	0.261 ± 0.014*	0.26 ± 0.024*	0.0001	28.3595
Carbohydrates; butanol biosynthesis	0.077 ± 0.005	0.084 ± 0.005	0.092 ± 0.008*	0.0257	11.9327
Carbohydrates; D-gluconate and ketogluconates metabolism	0.03 ± 0.007	0.052 ± 0.01*	0.06 ± 0.012*	0.0162	13.2444
Carbohydrates; fermentations in streptococci	0.078 ± 0.006	0.083 ± 0.009	0.101 ± 0.008*	<0.0001	29.8636
Carbohydrates; fructose utilization	0.103 ± 0.009	0.128 ± 0.011*	0.137 ± 0.016*	<0.0001	30.6238
Carbohydrates; glycerol and glycerol-3-phosphate uptake and utilization	0.171 ± 0.009	0.165 ± 0.015	0.194 ± 0.017*	0.001	20.6567
Carbohydrates; lactate utilization	0.038 ± 0.004	0.059 ± 0.005*	0.059 ± 0.005*	<0.0001	46.4617
Carbohydrates; L-ascorbate utilization (and related gene clusters)	0.022 ± 0.006	0.043 ± 0.009*	0.053 ± 0.011*	0.0017	19.1418
Carbohydrates; L-fucose utilization	0.069 ± 0.007	0.111 ± 0.014	0.107 ± 0.007*	0.0314	11.3576
Carbohydrates; maltose and maltodextrin utilization	0.227 ± 0.017	0.292 ± 0.019*	0.293 ± 0.031*	0.001	20.6068
Carbohydrates; mannitol utilization	0.032 ± 0.003	0.035 ± 0.004	0.045 ± 0.005*	0.0005	22.332
Carbohydrates; pyruvate:ferredoxin oxidoreductase	0.005 ± 0.001	0.002 ± 0.001	0.002 ± 0.001	<0.0001	27.64195
Carbohydrates; trehalose uptake and utilization	0.029 ± 0.004	0.041 ± 0.007	0.048 ± 0.009*	0.011	14.2553
Carbohydrates; xylose utilization	0.061 ± 0.005	0.081 ± 0.01*	0.084 ± 0.008*	0.0001	27.9072
Cell wall and capsule; capsular polysaccharides biosynthesis and assembly	0.074 ± 0.007	0.101 ± 0.007*	0.094 ± 0.007*	0.0257	11.9767
Cell wall and capsule; colanic acid biosynthesis	0.036 ± 0.006	0.063 ± 0.007*	0.067 ± 0.01*	<0.0001	32.7373
Cell wall and capsule; KDO2-lipid A biosynthesis	0.148 ± 0.009	0.208 ± 0.011*	0.194 ± 0.011*	0.0083	15.1438
Cell wall and capsule; lipid A-Ara4N pathway (Polymyxin resistance)	0.034 ± 0.005	0.053 ± 0.004*	0.054 ± 0.006*	0.0001	27.8501
Cell wall and capsule; lipopolysaccharide assembly	0.043 ± 0.003	0.067 ± 0.003*	0.061 ± 0.005*	0.0002	25.2526
Cell wall and capsule; LOS core oligosaccharide biosynthesis	0.053 ± 0.006	0.083 ± 0.011*	0.078 ± 0.01*	<0.0001	44.4746
Cofactors, Vitamins, prosthetic groups, pigments; menaquinone and phylloquinone biosynthesis	0.074 ± 0.006	0.119 ± 0.009*	0.107 ± 0.009*	0.0029	17.9292
**Dormancy and sporulation**	**0.186 ± 0.021**	**0.112 ± 0.015** *	**0.117 ± 0.016** *	**0.047**	**9.6622**
DNA metabolism; DNA repair, bacterial UmuCD system	0.025 ± 0.004	0.04 ± 0.003*	0.041 ± 0.004*	0.0002	25.0881
**Membrane transport**	**0.724 ± 0.043**	**0.817 ± 0.068** *	**0.865 ± 0.091** *	**0.0048**	**15.4806**
Membrane transport; ABC transporter oligopeptide (TC 3.A.1.5.1)	0.139 ± 0.008	0.1 ± 0.009*	0.134 ± 0.009	0.0017	19.1372
Membrane transport; ton and tol transport systems	0.09 ± 0.007	0.138 ± 0.006*	0.128 ± 0.01*	0.0002	24.4258
**Motility and chemotaxis**	**0.225 ± 0.028**	**0.32 ± 0.037** *	**0.365 ± 0.048** *	**0.0002**	**22.6026**
Motility and chemotaxis; bacterial chemotaxis	0.051 ± 0.01	0.076 ± 0.013	0.094 ± 0.017*	0.0078	15.3969
Motility and chemotaxis; flagellar motility	0.069 ± 0.006	0.091 ± 0.008*	0.1 ± 0.011*	<0.0001	34.0124
Motility and chemotaxis; flagellum	0.063 ± 0.012	0.096 ± 0.017	0.12 ± 0.019*	0.0058	16.365
**Nitrogen metabolism**	**0.19 ± 0.025**	**0.276 ± 0.04** *	**0.295 ± 0.059** *	**0.0169**	**16.2673**
Nitrogen metabolism; ammonia assimilation	0.034 ± 0.002	0.044 ± 0.004*	0.045 ± 0.005*	0.0001	26.1429
Nitrogen metabolism; nitrate and nitrite ammonification	0.083 ± 0.017	0.141 ± 0.026*	0.154 ± 0.038*	0.0126	13.9455
Phages, prophages, transposable elements, plasmids; T4-like non-cyanophage core proteins	0.052 ± 0.008	0.014 ± 0.004*	0.045 ± 0.021	0.0061	16.1775
Phages, prophages, transposable elements; phage capsid proteins	0.088 ± 0.014	0.035 ± 0.005*	0.052 ± 0.01*	0.0002	25.5776
Phosphorus metabolism; alkylphosphonate utilization	0.025 ± 0.006	0.044 ± 0.009	0.052 ± 0.013*	0.033	11.1529
**Potassium metabolism**	**0.447 ± 0.019**	**0.502 ± 0.016** *	**0.51 ± 0.024** *	**0.0048**	**15.4806**
Potassium metabolism; glutathione-regulated potassium-efflux system and associated functions	0.189 ± 0.008	0.218 ± 0.009	0.225 ± 0.011*	0.0397	10.6399
Protein metabolism; peptidyl-prolyl cis-trans isomerase	0.034 ± 0.003	0.056 ± 0.003*	0.053 ± 0.004*	0.0001	27.0287
Protein metabolism; periplasmic disulfide interchange	0.033 ± 0.003	0.051 ± 0.003*	0.046 ± 0.004*	0.0064	15.978
**Regulation and cell signaling**	**0.332 ± 0.025**	**0.406 ± 0.035** *	**0.434 ± 0.047** *	**<0.0001**	**28.3902**
Regulation and cell signaling; Orphan regulatory proteins	0.03 ± 0.007	0.057 ± 0.012	0.061 ± 0.015*	0.0322	11.2595
Regulation and cell signaling; stringent response, (p)ppGpp metabolism	0.036 ± 0.002	0.052 ± 0.003*	0.046 ± 0.002	0.0462	10.2449
**Respiration**	**0.745 ± 0.037**	**0.85 ± 0.054** *	**0.872 ± 0.087** *	**<0.0001**	**36.2003**
Respiration; ATP synthases HGM	0.111 ± 0.014	0.042 ± 0.008*	0.052 ± 0.01*	0.0052	16.6042
Respiration; formate dehydrogenase	0.023 ± 0.006	0.045 ± 0.008*	0.047 ± 0.011*	0.009	14.8436
Respiration; respiration/HGM	0.08 ± 0.008	0.124 ± 0.01*	0.128 ± 0.015*	<0.0001	44.6248
Respiration; H2:CoM-S-S-HTP oxidoreductase	0.014 ± 0.01	0.007 ± 0.001*	0.006 ± 0.001*	0.0012	20.3934
Respiration; carbon monoxide induced hydrogenase	0.021 ± 0.002	0.009 ± 0.001*	0.007 ± 0.001*	0.0002	24.384
**Stress response**	**0.471 ± 0.031**	**0.54 ± 0.053**	**0.577 ± 0.072** *	**0.0144**	**12.7939**
Stress response; carbon starvation	0.048 ± 0.003	0.056 ± 0.004*	0.057 ± 0.007*	0.0177	12.9543
Stress response; choline and betaine uptake and betaine biosynthesis	0.035 ± 0.004	0.044 ± 0.006	0.054 ± 0.008*	0.001	20.643
**Sulfur metabolism**	**0.085 ± 0.005**	**0.105 ± 0.007** *	**0.114 ± 0.011** *	**<0.0001**	**60.6093**
Sulfur metabolism; alkanesulfonate assimilation	0.036 ± 0.003	0.049 ± 0.004*	0.052 ± 0.006*	<0.0001	45.9098
**Virulence**	**0.457 ± 0.046**	**0.613 ± 0.077** *	**0.659 ± 0.12** *	**0.0144**	**12.9433**
Virulence; Multidrug efflux pump in campylobacter jejuni (CmeABC operon)	0.026 ± 0.001	0.041 ± 0.003*	0.039 ± 0.003*	<0.0001	30.7113
Virulence; multidrug resistance, Tripartite systems found in gram negative bacteria	0.044 ± 0.004	0.074 ± 0.004*	0.069 ± 0.007*	0.0029	17.9578

Mean percent ± standard error of means. *Indicates significantly different to Control (ANCOM-BC *Q* < 0.05).

Other differences included genes involved in amino acid metabolism, nitrogen metabolism, cell wall and capsule related genes, cell motility, and virulence, all of which in general were more abundant in the cecal microbiome of piglets fed HN001. Further differences included genes related to sulfur metabolism, vitamin K metabolism (menaquinone and phylloquinone biosynthesis), and choline and betaine metabolism, which were also significantly (*Q* < 0.05) more abundant in HN001 fed piglets. Finally, other gene categories that differed included phage related genes, which were less abundant in HN001 groups.

#### Fecal microbiome

**FIGURE 3:**
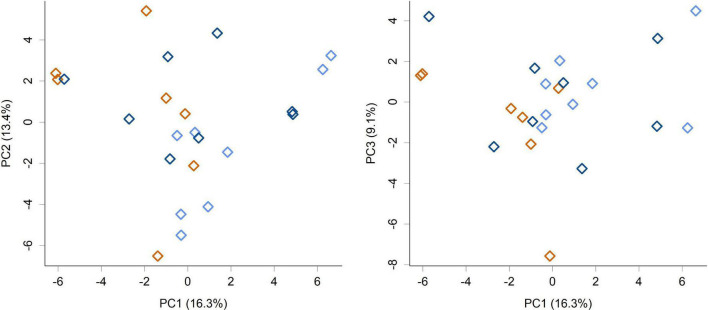
PCA scores plot of the piglet fecal microbiome community composition at the genus level (PC1 vs. PC2 and PC1 vs. PC3 shown). Colors indicate groups; Control (orange), HN001 Low (light blue), HN001 High (dark blue). Permutation MANOVA *P* = 0.07.

**FIGURE 4:**
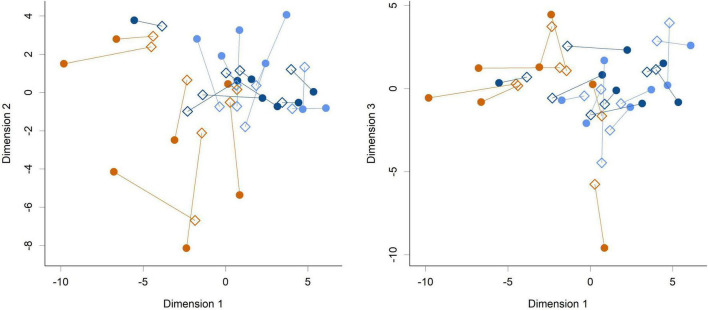
Procrustes rotation analysis of the cecal (circles) and fecal (diamonds) communities at the genus level (Dimension 1 vs. Dimension 2 and Dimension 1 vs. Dimension 3 shown). Colors indicate groups; Control (orange), HN001 Low (light blue), HN001 High (dark blue). Lines join fecal and cecal communities from the same piglet. Procrustes symmetric rotation correlation = 0.704 (*P* < 0.001).

While feeding *L. rhamnosus* HN001 clearly impacted the cecal microbiome, the effects were much less noticeable in the feces. Fecal communities did not display conspicuous separation between groups by PCA (Figure 3) and permutation MANOVA only trended toward significance (*P* = 0.07). Furthermore, at the genus level, no significant differences were found between groups (FDR > 0.4). Although fecal communities did not appear to differentiate by treatment unlike in the cecum, fecal community composition did correlate with the cecal community from the same piglet. Procrustes rotation analysis showed a significant correlation (*r* = 0.7, *P* < 0.001) between PCA projections of the cecal and fecal microbial communities (Figure 4).

### Gut tissue transcriptome

The median number of paired-end reads per sample following quality trimming was 13.4 M, with a standard deviation, minimum, and maximum number of reads of 1.9, 10.4, and 18.3 M reads, respectively.

**FIGURE 5:**
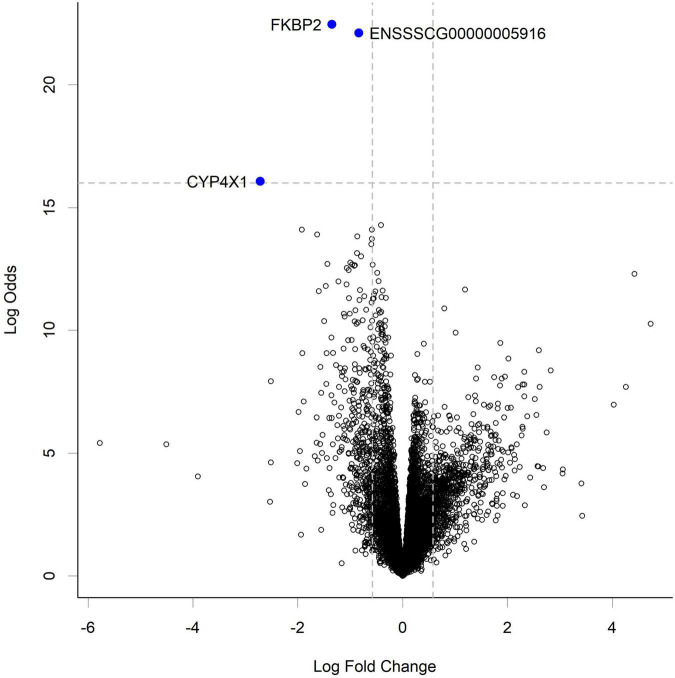
Volcano plot showing gene expression change in the cecum from piglets in the HN001 High group compared to Controls. Transcripts considered differentially expressed are those with FDR < 0.05 (above horizontal dashed line) and fold change > | 1.5x|, which equates to a log fold change > | 0.58| (indicated by vertical dashed lines). A negative log fold change indicates lower expression in the HN001 High group whereas a positive log fold change indicates a high expression in the HN001 High group compared to Controls.

RNA-seq analysis of the gut tissue transcriptome showed changes in expression of 3 transcripts at the highest HN001 dose in the cecum (Figure 5). Of these genes, 2 had known functions; *FKBP2* (Ensembl ID ENSSSCG00000033757) and *CYP4* × *1* (Ensembl ID ENSSSCG00000031778). *FKBP2*, which encodes FK506 Binding Protein 2, had a 2.6-fold lower expression in the HN001 High group compared to Controls (FDR = 0.001). Similarly, expression of *CYP4* × *1*, which encodes one of the Cytochrome P450 family of enzymes, was also downregulated in the HN001 High group compared to Controls (6.6-fold lower, FDR = 0.001).

**FIGURE 6:**
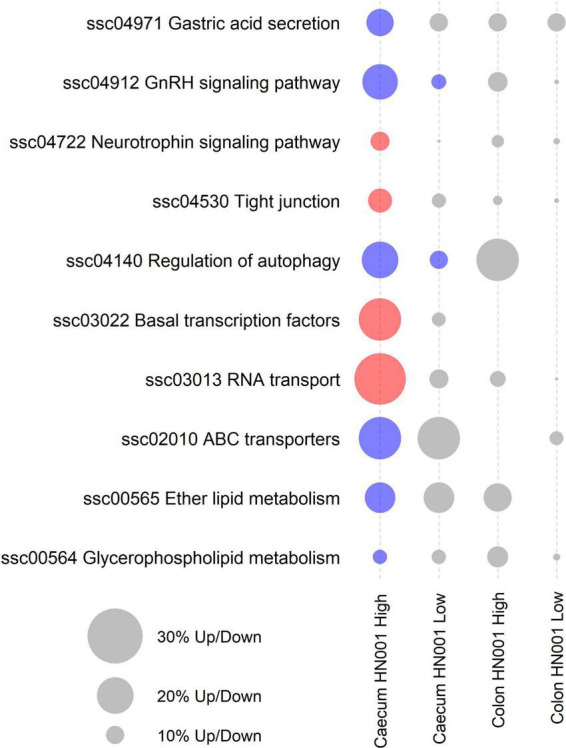
KEGG pathways differentially expressed by GSEA (*P* < 0.05) in at least one treatment and tissue. Red circles indicate overall significantly higher expression compared to Controls and blue circles indicate overall significantly lower expression compared to Controls. Gray circles indicate pathway not differentially expressed (*P* > 0.05). Size of circle proportional to the number of genes up or down regulated.

While at the per gene level *L. rhamnosus* HN001 only modified the expression of three genes and only in the HN001 High group, GSEA identified 10 KEGG pathways that were differentially expressed (*P* < 0.05) in the cecum of piglets in the HN001 High group compared to the Control group, and these two pathways were also differentially expressed in the HN001 Low group (Figure 6).

**FIGURE 7:**
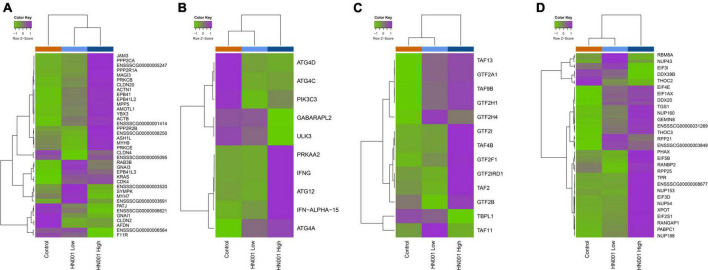
Heatmaps showing mean expression profiles and hierarchical clustering of genes within the KEGG **(A)** *tight junction*, **(B)** *regulation of autophagy*, **(C)** *basal transcription factors*, and **(D)** *RNA transport* pathways. Color ribbon across top of heatmap indicates sample treatment group; Control (orange), HN001 Low (light blue), and HN001 High (dark blue).

Analysis of the collective gene expression profiles in each of these KEGG pathways by permutation ANOVA also confirmed the overall significant changes in expression patterns in four pathways between the HN001 High and the Control groups; *Tight junction* (ssc04530), *Basal transcription factors* (ssc03022), and *RNA transport* (ssc03013) pathways were more highly expressed in the HN001 High group (*P* = 0.005, 0.034, and 0.008, respectively), whereas the *Regulation of autophagy* (ssc04140) pathway showed higher expression in the Control group (*P* = 0.008). Although expression of KEGG pathways were not significantly altered in the HN001 Low group compared to Controls, with the exception of the GnRH signaling pathway (ssc04912) and Regulation of autophagy (ssc04140) pathway, expression profile patterns were generally intermediate between Controls and the HN001 High group piglets (Figure 7). Consistent with the microbiome results where *L. rhamnosus* HN001 significantly altered the cecal microbiome but only had minor effects on the fecal microbiome, feeding *L. rhamnosus* HN001 did not lead to any genes passing the thresholds for differential expression in the colon, nor did GSEA show any differentially expressed KEGG pathways.

## Discussion

In this study we show that supplementation with *L. rhamnosus* HN001 can have dramatic effects on the microbiome of the cecum in a piglet model, with broad changes in relative abundance of the dominant taxa. At the same time, significant effects on the fecal microbiome were not observed. Associated with the change in cecal microbiota composition, we showed *L. rhamnosus* HN001 also altered the cecal tissue transcriptome compared to controls. However, this effect was not observed in the colon tissue transcriptome, which is consistent with our results from the fecal microbiome.

Supplementation with *L. rhamnosus* HN001 at the highest dose led to increased proportions of *L. rhamnosus* in the cecum and in the feces, as expected. However, *L. rhamnosus* HN001 at the lower dose did not lead to a significant increase. Similarly, the relative abundance of the wider *Lactobacillus* genus overall was increased in the HN001 High group. Interestingly, the relative abundance of the *Lactobacillus* genus was substantially higher than that of *L. rhamnosus* itself (over 40-fold higher). Because taxonomic identification to the species level is not always reliable from either 16S rRNA sequences or shotgun metagenome data (33, 34), it is uncertain whether the increased *Lactobacillus* was due to increased sequences originating from *L. rhamnosus* that could not be classified to the species level due to factors such as read length and alignment position, or whether it was from increases in *Lactobacillus* of different species. *Lactobacillus* was present in the cecum of control piglets, indicating the presence of this genus as an autochthonous member of the gut microbial community.

While cecal *Lactobacillus* proportions increased from around 3% in control piglets to around 6% in the HN001 high group, greater changes, both in terms of fold change and relative abundance, were observed in a wide range of taxa. *Bacteroidetes* in the cecum were increased by both low and high doses of HN001 and within the *Bacteroidetes*, the most prominent change occurred in *Prevotella*, which increased over threefold on supplementation with HN001. Despite being a prominent member of the human gastrointestinal microbiome, the role of *Prevotella* is still relatively unclear. There is evidence for *Prevotella* as a biomarker for diets high in plant fiber (35, 36), and increased *Prevotella* has been associated with improved glucose response (37) and reduction in body fat (38). Conversely, a reduction in *Prevotella* has been associated with increased behavioral problems in infants at 2 years of age (39). In contrast, there are also studies that show increased *Prevotella* can exacerbate intestinal inflammation and autoimmune disease (40–42). However, in the absence of an underlying health issue, the balance of the evidence points to *Prevotella* being an important member of the gut community that contributes to polysaccharide breakdown and SCFA production (43). Indeed, the increase in abundance of genes related to utilization of polysaccharides such as xylose, trehalose, and fucose in HN001 groups is probably reflective of the increase in *Prevotella* (44–46). The most likely polysaccharide substrates relevant to the piglets in our study are likely to be host-derived glycans, such as mucin and lactose, which can be metabolized by different species of *Prevotella* (47–49).

While it is unclear why supplementation with HN001, a *Lacticaseibacillus*, would stimulate an increase in *Prevotella*, a previous study has shown that bacteriocin-producing *Ligilactobacillus salivarius* increased intestinal *Prevotella* in mice, an effect which was not observed with *L. salivarius* lacking the specific bacteriocin gene (50). Bacteriocins produced by *L. rhamnosus* HN001 appear to have a limited spectrum and are only active against some Gram positive bacteria (51), which could be providing a competitive advantage for *Prevotella*, leading to their increase in relative abundance.

Other changes in the cecal microbiome included a significant decrease in *Methanobrevibacter* in the HN001 groups. In the gut, *Methanobrevibacter* primarily converts CO_2_ and H_2_ to form methane, although formate can also be used as substrate for methanogenesis (52). For methanogens, an important limiting factor is the concentration of H_2_, which in the gut is mainly produced by microbial fermentation of carbohydrates (53). Interestingly, the use of lactic acid bacteria has been proposed for reducing methane production in ruminants, with one of the hypothesized mechanisms of action being the promotion of lactate-utilizing microbes and shifting microbial fermentation toward pathways that reduce the formation of hydrogen (54). Supporting this theory, we found genes involved in lactate utilization were increased in the cecum community of piglets supplemented with HN001. Furthermore, free hydrogen appears to be produced primarily by the *Firmicutes* (55), which were significantly reduced in piglets supplemented with HN001. Other differences that indicate pathways involved in carbohydrate fermentation and hydrogen utilization are altered by HN001 include changes in gene abundances related to methanogen metabolism, such as the SEED functions *Carbon monoxide induced hydrogenase, H2:CoM-S-S-HTP oxidoreductase*, and *Aromatic amino acid interconversions with aryl acids*. Carbon monoxide induced hydrogenase genes can be used by methanogens to use methyl groups as a substrate (56), while reduction of heterodisulfide (CoM-S-S-HTP) is a key energy metabolism pathway in methanogenic Archaea (57). Interestingly, the SEED function *Aromatic amino acid interconversions with aryl acids*, an important pathway used by methanogens to transform aromatic amino acids to aryl amino acids (58), was also reduced in both HN001 groups compared to Controls. Another factor which may impact the relationship between hydrogen availability and methane production are formate dehydrogenase genes, which convert formate to CO_2_ and H_2_. Genes assigned to this function were more abundant in the HN001 groups.

The cecal microbiomes of HN001 fed piglets were also characterized by a noticeable increase in the abundance of genes related to amino acid, nitrogen, and protein metabolism. A wide variety of gut microbes are able to decarboxylate amino acids to produce an amine plus carbon dioxide, and of these, lactobacilli and Proteobacteria are prominent representatives (59, 60), both of which were also increased following HN001 supplementation. Further conversion steps following decarboxylation of amino acids can lead to a variety of end products, but the most abundant are actually SCFAs (60). This is an important point as fermentation of amino acids and proteins is often assumed to lead to toxic compounds, and therefore negative impacts on the host. However, there is no evidence of any such toxicity effects arising from HN001. The metagenomes from piglets fed *L. rhamnosus* HN001 were also characterized by a prominent increase in the relative abundance of genes relating to K group vitamins. Lactobacilli have been shown to produce vitamin K (61, 62), which is a plausible explanation for the differences observed in these pathways. Changes in vitamin biosynthesis in the gut may also have wider impacts on the microbiome ecosystem as many commensal bacteria depend on the supply of vitamins through cross-feeding, including prominent butyrate producing members of the Firmicutes, such as *Faecalibacterium prausnitzii* (63). Together, these observations may explain how the dietary supplementation of a single lactic acid-producing bacterium, *L. rhamnosus* HN001, can lead to such dramatic changes in the overall microbiome composition, by influencing microbiome carbohydrate fermentation, amino acid metabolism, and vitamin availability.

In addition to the extensive alterations in the microbial community composition of the cecum, supplementation with *L. rhamnosus* HN001 also led to changes in gene expression profiles in the cecal tissue. Although analysis at the gene level only revealed two differentially expressed genes with known functions, GSEA highlighted several differentially expressed pathways. Overall expression of genes involved in tight junction formation and activity were more highly expressed in the cecum of piglets receiving the higher dose of *L. rhamnosus* HN001, compared to Controls. Tight junction proteins are important molecules that strongly influence intestinal barrier integrity, and perturbations in this barrier can lead to inflammation and serious disorders in the gastrointestinal tract and other parts of the body (64). Previous studies have shown that *L. rhamnosus* HN001 can beneficially enhance intestinal barrier integrity, both in cell (9) and animal models (65). Treatment with *L. rhamnosus* HN001 in animal models has also been shown to decrease the severity of necrotizing enterocolitis, which is characterized by extensive destruction of the intestinal epithelial cell layer (6). In this instance, the protective effects of HN001 were modulated by the activation of Toll-like receptor 9 (6), a receptor for microbial DNA expressed in immune system cells including dendritic cells, and other antigen presenting cells (66).

Related to the effects on tight junction gene expression, *L. rhamnosus* HN001 also altered expression in pathways related to neutrophin signaling and autophagy. The neurotrophic factor glial cell-derived neurotrophic factor (GDNF) has been shown to attenuate inflammation by increasing expression of tight junction proteins in a mouse model (67). Increased expression of another neurotrophic factor, brain-derived neurotrophic factor (BDNF), has also been shown to suppress autophagy in mice (68). In our study, expression of neutrophin signaling pathways in the cecal tissue was increased by *L. rhamnosus* HN001 at the high dose, while expression of autophagy pathways was decreased. Autophagy is a cellular process controlling the ordered removal of dysfunctional cells, which plays a major role in the regulation of inflammation (69). Direct links between autophagy proteins and tight junction integrity has also been demonstrated in previous studies. For example, autophagy-related protein-6 (ATG6) has been shown to disrupt tight junction integrity in a cell model by promoting the endocytosis of the tight junction protein occludin (70), while in a rat model of intestinal inflammation, decreased autophagy was associated with upregulation of claudin-2 (71), another tight junction protein. These studies and our results highlight how inflammation, intestinal barrier function and the intestinal microbes, whether resident or introduced, are interconnected.

Our study also highlights the importance for considering the specific location in the gastrointestinal tract when studying the microbiome; almost all microbiome studies in humans use fecal samples as a proxy for the microbial communities within the gut. While using fecal samples is usually the only option due to the difficulty of accessing the communities within the gut of a healthy human subject, our study shows that care must be taken when interpreting the results because they may not adequately reflect any differences seen within the entire gastrointestinal tract.

## Conclusion

Supplementation of infant formula with *L. rhamnosus* HN001 can have dramatic effects on the developing cecal microbiome in a piglet model. The changes observed could not be explained simply by the expansion of the *Lactobacillus* genus as the magnitude of differences in other taxa was greater than the magnitude of *Lactobacillus* increase in the HN001 supplemented groups. The differences in taxonomic composition and relative abundances of gene functions in the cecum suggests *L. rhamnosus* HN001-induced changes in the microbiome included alterations in carbohydrate, hydrogen, methane, and amino acid metabolism. Concomitant with alterations in the cecal microbiome, host gene expression profiles in the cecal tissue were also impacted by *L. rhamnosus* HN001 supplementation. Changes in host gene expression induced by *L. rhamnosus* HN001 is consistent with improved intestinal barrier integrity and decreased inflammation. While *L. rhamnosus* HN001-induced changes in the cecal microbiome and cecal tissue gene expression were apparent, differences in the colonic tissue gene expression and fecal microbiome were less distinct. This work shows *L. rhamnosus* HN001 can influence the microbiome and host physiology, with changes that are likely to be beneficial to the host. While our study has shown *L. rhamnosus* HN001 impacts the cecal microbiome composition and metagenome, future studies could gain further insights by investigating the microbiome meta-transcriptome and metabolome to better understand the activity of the microbiome. Finally, the translation of animal model results, such as ours, to humans requires careful attention and confirmation in follow up studies.

## Data availability statement

The datasets presented in this study can be found in online repositories. The names of the repository/repositories and accession number(s) can be found below: https://www.ncbi.nlm.nih.gov/, PRJNA823879.

## Ethics statement

The animal study was reviewed and approved by the AgResearch Limited (Grasslands) Animal Ethics Committee (Ethics Approval No. 13982).

## Author contributions

NR, RD, JD, NH, RA, and WY designed the experiments. LR, JP, KA, HD, and MG performed the animal experiments and DNA and RNA extractions. PM and WY carried out bioinformatics and statistical analyses. WY wrote the manuscript with contributions from all authors. All authors approved the final version of the manuscript.

## References

[1] Joint FAO/WHO Working Group. *Probiotics in Food: Health and Nutritional Properties and Guidelines for Evaluation.* Geneva: World Health Organization (2006).

[2] Braegger C Chmielewska A Decsi T Kolacek S Mihatsch W Moreno L Supplementation of infant formula with probiotics and/or prebiotics: a systematic review and comment by the ESPGHAN committee on nutrition. *J Pediatr Gastroenterol Nutr.* (2011) 52:238–50. 21150647 10.1097/MPG.0b013e3181fb9e80

[3] Wickens K Barthow C Mitchell EA Stanley TV Purdie G Rowden J Maternal supplementation alone with *Lactobacillus rhamnosus* HN001 during pregnancy and breastfeeding does not reduce infant eczema. *Pediatr Allergy Immunol.* (2018) 29:296–302. 10.1111/pai.12874 29415330

[4] Slykerman RF Hood F Wickens K Thompson JMD Barthow C Murphy R Effect of *Lactobacillus rhamnosus* HN001 in pregnancy on postpartum symptoms of depression and anxiety: a randomised double-blind placebo-controlled trial. *EBioMedicine.* (2017) 24:159–65. 10.1016/j.ebiom.2017.09.013 28943228 PMC5652021

[5] Wickens K Barthow C Mitchell EA Kang J van Zyl N Purdie G Effects of *Lactobacillus rhamnosus* HN001 in early life on the cumulative prevalence of allergic disease to 11 years. *Pediatr Allergy Immunol.* (2018) 29:808–14. 10.1111/pai.12982 30430649

[6] Good M Sodhi CP Ozolek JA Buck RH Goehring KC Thomas DL *Lactobacillus rhamnosus* HN001 decreases the severity of necrotizing enterocolitis in neonatal mice and preterm piglets: evidence in mice for a role of TLR9. *Am J Physiol Gastrointest Liver Physiol.* (2014) 306:G1021–32. 24742987 10.1152/ajpgi.00452.2013 PMC4042115

[7] Tannock GW Munro K Harmsen HJ Welling GW Smart J Gopal PK. Analysis of the fecal microflora of human subjects consuming a probiotic product containing *Lactobacillus rhamnosus* DR20. *Appl Environ Microbiol.* (2000) 66:2578–88. 10.1128/AEM.66.6.2578-2588.2000 10831441 PMC110584

[8] Murphy R Morgan XC Wang XY Wickens K Purdie G Fitzharris P Eczema-protective probiotic alters infant gut microbiome functional capacity but not composition: sub-sample analysis from a RCT. *Benef Microb.* (2019) 10:5–17. 10.3920/BM2017.0191 30574802

[9] Anderson RC Cookson AL McNabb WC Kelly WJ Roy NC. *Lactobacillus plantarum* DSM 2648 is a potential probiotic that enhances intestinal barrier function. *FEMS Microbiol Lett.* (2010) 309:184–92. 10.1111/j.1574-6968.2010.02038.x 20618863

[10] Ulluwishewa D Anderson RC Young W McNabb WC van Baarlen P Moughan PJ Live faecalibacterium prausnitzii in an apical anaerobic model of the intestinal epithelial barrier. *Cell Microbiol.* (2015) 17:226–40. 10.1111/cmi.12360 25224879

[11] Villena J Aso H Kitazawa H. Regulation of toll-like receptors-mediated inflammation by immunobiotics in bovine intestinal epitheliocytes: role of signaling pathways and negative Regulators. *Front Immunol.* (2014) 5:421. 10.3389/fimmu.2014.00421 25228903 PMC4151153

[12] Moens F Van den Abbeele P Basit AW Dodoo C Chatterjee R Smith B A four-strain probiotic exerts positive immunomodulatory effects by enhancing colonic butyrate production in vitro. *Int J Pharm.* (2019) 555:1–10. 10.1016/j.ijpharm.2018.11.020 30445175

[13] Zmora N Zilberman-Schapira G Suez J Mor U Dori-Bachash M Bashiardes S Personalized gut mucosal colonization resistance to empiric probiotics is associated with unique host and microbiome features. *Cell.* (2018) 174:1388–405.e21. 10.1016/j.cell.2018.08.041 30193112

[14] Hou Q Zhao F Liu W Lv R Khine WWT Han J Probiotic-directed modulation of gut microbiota is basal microbiome dependent. *Gut Microbes.* (2020) 12:1736974. 10.1080/19490976.2020.1736974 32200683 PMC7524168

[15] Lavelle A Lennon G O’Sullivan O Docherty N Balfe A Maguire A Spatial variation of the colonic microbiota in patients with ulcerative colitis and control volunteers. *Gut.* (2015) 64:1553–61. 10.1136/gutjnl-2014-307873 25596182 PMC4602252

[16] Puiman P Stoll B. Animal models to study neonatal nutrition in humans. *Curr Opin Clin Nutr Metab Care.* (2008) 11:601–6. 10.1097/MCO.0b013e32830b5b15 18685456

[17] Calder PC Krauss-Etschmann S de Jong EC Dupont C Frick JS Frokiaer H Early nutrition and immunity - progress and perspectives. *Br J Nutr.* (2006) 96:774–90. 17010239

[18] Fil JE Joung S Hayes CA Dilger RN. Influence of rearing environment on longitudinal brain development, object recognition memory, and exploratory behaviors in the domestic pig (*Sus scrofa*). *Front Neurosci.* (2021) 15:649536. 10.3389/fnins.2021.649536 33841090 PMC8024486

[19] Food Standards Australia New Zealand. Microbiology risk assessment: L-lactic acid producing microorganisms. *Proceedings of the Safety & Food Technology Consultation Paper Proposal p1028–Review of the Regulation of Infant Formula Products.* Wellington: Food Standards Australia New Zealand (FSANZ) (2021). P. 156–21.

[20] Bolger AM Lohse M Usadel B. Trimmomatic: a flexible trimmer for Illumina sequence data. *Bioinformatics.* (2014) 30:2114–20. 10.1093/bioinformatics/btu170 24695404 PMC4103590

[21] Zhang J Kobert K Flouri T Stamatakis A. PEAR: a fast and accurate illumina paired-end reAd mergeR. *Bioinformatics.* (2014) 30:614–20. 10.1093/bioinformatics/btt593 24142950 PMC3933873

[22] Bushnell B. *(Sponsor Org.: USDOE Office of Science (SC)).* Berkeley, CA: U.S. Department of Energy (2021).

[23] Buchfink B Xie C Huson DH. Fast and sensitive protein alignment using diamond. *Nat Methods.* (2015) 12:59–60. 10.1038/nmeth.3176 25402007

[24] Huson DH Beier S Flade I Górska A El-Hadidi M Mitra S Megan community edition - interactive exploration and analysis of large-scale microbiome sequencing data. *PLoS Comput Biol.* (2016) 12:e1004957. 10.1371/journal.pcbi.1004957 27327495 PMC4915700

[25] Overbeek R Begley T Butler RM Choudhuri JV Chuang HY Cohoon M The subsystems approach to genome annotation and its use in the project to annotate 1000 genomes. *Nucleic Acids Res.* (2005) 33:5691–702. 10.1093/nar/gki866 16214803 PMC1251668

[26] Dixon P. Vegan, a package of R functions for community ecology. *J Veg Sci.* (2003) 14:927–30. 10.1111/j.1654-1103.2003.tb02228.x

[27] Lin H Peddada SD. Analysis of compositions of microbiomes with bias correction. *Nat Commun.* (2020) 11:3514. 10.1038/s41467-020-17041-7 32665548 PMC7360769

[28] Dobin A Davis CA Schlesinger F Drenkow J Zaleski C Jha S Star: ultrafast universal RNA-seq aligner. *Bioinformatics.* (2013) 29:15–21. 10.1093/bioinformatics/bts635 23104886 PMC3530905

[29] Robinson MD McCarthy DJ Smyth GK. edgeR: a bioconductor package for differential expression analysis of digital gene expression data. *Bioinformatics.* (2010) 26:139–40. 10.1093/bioinformatics/btp616 19910308 PMC2796818

[30] Smyth GK. Linear models and empirical bayes methods for assessing differential expression in microarray experiments. *Stat Appl Genet Mol Biol.* (2004) 3:3. 10.2202/1544-6115.1027 16646809

[31] Kanehisa M Goto S. KEGG: kyoto encyclopedia of genes and genomes. *Nucleic Acids Res.* (2000) 28:27–30. 10.1093/nar/28.1.27 10592173 PMC102409

[32] Carlson M. *KEGG.db: A Set of Annotation Maps for KEGG. R Package Version 3.2.3.* (2016).

[33] Peabody MA Van Rossum T Lo R Brinkman FSL. Evaluation of shotgun metagenomics sequence classification methods using in silico and in vitro simulated communities. *BMC Bioinform.* (2015) 16:362. 10.1186/s12859-015-0788-5 26537885 PMC4634789

[34] Johnson JS Spakowicz DJ Hong BY Petersen LM Demkowicz P Chen L Evaluation of 16S rRNA gene sequencing for species and strain-level microbiome analysis. *Nat Commun.* (2019) 10:5029. 10.1038/s41467-019-13036-1 31695033 PMC6834636

[35] De Filippo C Cavalieri D Di Paola M Ramazzotti M Poullet JB Massart S Impact of diet in shaping gut microbiota revealed by a comparative study in children from Europe and rural Africa. *Proc Natl Acad Sci U S A.* (2010) 107:14691–6. 10.1073/pnas.1005963107 20679230 PMC2930426

[36] De Filippis F Pasolli E Tett A Tarallo S Naccarati A De Angelis M Distinct genetic and functional traits of human intestinal prevotella copri strains are associated with different habitual diets. *Cell Host Microbe.* (2019) 25:444–53.e3. 10.1016/j.chom.2019.01.004 30799264

[37] Kovatcheva-Datchary P Nilsson A Akrami R Lee YS De Vadder F Arora T Dietary fiber-induced improvement in glucose metabolism is associated with increased abundance of prevotella. *Cell Metab.* (2015) 22:971–82. 10.1016/j.cmet.2015.10.001 26552345

[38] Hjorth MF Blædel T Bendtsen LQ Lorenzen JK Holm JB Kiilerich P Prevotella-to-*Bacteroides* ratio predicts body weight and fat loss success on 24-week diets varying in macronutrient composition and dietary fiber: results from a post-hoc analysis. *Int J Obes.* (2018) 43:149–57. 10.1038/s41366-018-0093-2 29777234 PMC6331389

[39] Loughman A Ponsonby AL O’Hely M Symeonides C Collier F Tang MLK Gut microbiota composition during infancy and subsequent behavioural outcomes. *EBioMedicine.* (2020) 52:102640. 10.1016/j.ebiom.2020.102640 32062351 PMC7016366

[40] Scher JU Sczesnak A Longman RS Segata N Ubeda C Bielski C Expansion of intestinal *Prevotella* copri correlates with enhanced susceptibility to arthritis. *eLife.* (2013) 2:e01202. 10.7554/eLife.01202 24192039 PMC3816614

[41] Iljazovic A Roy U Gálvez EJC Lesker TR Zhao B Gronow A Perturbation of the gut microbiome by *Prevotella* spp. enhances host susceptibility to mucosal inflammation. *Mucosal Immunol.* (2020) 14:1–12. 10.1038/s41385-020-0296-4 32433514 PMC7790746

[42] Leite AZ Rodrigues NC Gonzaga MI Paiolo JCC de Souza CA Stefanutto NAV Detection of increased plasma interleukin-6 levels and prevalence of prevotella copri and *Bacteroides* vulgatus in the feces of type 2 diabetes patients. *Front Immunol.* (2017) 8:1107. 10.3389/fimmu.2017.01107 28966614 PMC5605568

[43] Precup G Vodnar D-C. Gut *Prevotella* as a possible biomarker of diet and its eubiotic versus dysbiotic roles: a comprehensive literature review. *Br J Nutr.* (2019) 122:131–40. 10.1017/S0007114519000680 30924428

[44] Linares-Pastén JA Hero JS Pisa JH Teixeira C Nyman M Adlercreutz P Novel xylan-degrading enzymes from polysaccharide utilizing loci of *Prevotella* copri DSM18205. *Glycobiology.* (2021) 31:1330–49. 10.1093/glycob/cwab056 34142143 PMC8631079

[45] Gálvez EJC Iljazovic A Amend L Lesker TR Renault T Thiemann S Distinct polysaccharide utilization determines interspecies competition between intestinal *Prevotella* spp. *Cell Host Microbe.* (2020) 28:838–52.e6. 10.1016/j.chom.2020.09.012 33113351

[46] Fehlner-Peach H Magnabosco C Raghavan V Scher JU Tett A Cox LM Distinct polysaccharide utilization profiles of human intestinal *Prevotella* copri isolates. *Cell Host Microbe.* (2019) 26:680–90.e5. 10.1016/j.chom.2019.10.013 31726030 PMC7039456

[47] Efimov BA Chaplin AV Shcherbakova VA Suzina NE Podoprigora IV Shkoporov AN. *Prevotella* rara sp. nov., isolated from human faeces. *Int J Syst Evol Microbiol.* (2018) 68:3818–25. 10.1099/ijsem.0.003066 30339117

[48] Roux V Robert C Raoult D. Non-contiguous finished genome sequence of *Prevotella* timonensis type strain 4401737(T.). *Stand Genomic Sci.* (2014) 9:1344–51. 10.4056/sigs.5098948 25197502 PMC4148998

[49] Wright DP Rosendale DI Robertson AM. *Prevotella* enzymes involved in mucin oligosaccharide degradation and evidence for a small operon of genes expressed during growth on mucin. *FEMS Microbiol Lett.* (2000) 190:73–9. 10.1111/j.1574-6968.2000.tb09265.x 10981693

[50] Riboulet-Bisson E Sturme MH Jeffery IB O’Donnell MM Neville BA Forde BM Effect of lactobacillus salivarius bacteriocin abp118 on the mouse and pig intestinal microbiota. *PLoS One.* (2012) 7:e31113. 10.1371/journal.pone.0031113 22363561 PMC3281923

[51] Aguilar-Uscanga BR Solís-Pacheco JR Plascencia L Aguilar-Uscanga MG García HS Lacroix M. Effect of culture medium on bacteriocin production by *Lactobacillus rhamnosus* HN001 and Lactobacillus reuteri ATCC 53608. *J Microbiol Biotechnol Food Sci.* (2013) 2:2462–8.

[52] Kelly WJ Mackie RI Attwood GT Janssen PH McAllister TA Leahy SC. Hydrogen and formate production and utilisation in the rumen and the human colon. *Anim Microbiome.* (2022) 4:22. 10.1186/s42523-022-00174-z 35287765 PMC8919644

[53] Flint HJ Scott KP Duncan SH Louis P Forano E. Microbial degradation of complex carbohydrates in the gut. *Gut Microb.* (2012) 3:289–306. 10.4161/gmic.19897 22572875 PMC3463488

[54] Doyle N Mbandlwa P Kelly WJ Attwood G Li Y Ross RP Use of lactic acid bacteria to reduce methane production in ruminants, a critical review. *Front Microbiol.* (2019) 10:2207. 10.3389/fmicb.2019.02207 31632365 PMC6781651

[55] Carbonero F Benefiel AC Gaskins HR. Contributions of the microbial hydrogen economy to colonic homeostasis. *Nat Rev Gastroenterol Hepatol.* (2012) 9:504–18. 10.1038/nrgastro.2012.85 22585131

[56] Daniels L Fuchs G Thauer RK Zeikus JG. Carbon monoxide oxidation by methanogenic bacteria. *J Bacteriol.* (1977) 132:118–26. 21159 10.1128/jb.132.1.118-126.1977 PMC221834

[57] Setzke E Hedderich R Heiden S Thauer RK. H2: heterodisulfide oxidoreductase complex from *Methanobacterium* thermoautotrophicum. Composition and properties. *Eur J Biochem.* (1994) 220:139–48. 10.1111/j.1432-1033.1994.tb18608.x 8119281

[58] Porat I Waters BW Teng Q Whitman WB. Two biosynthetic pathways for aromatic amino acids in the archaeon *Methanococcus* maripaludis. *J Bacteriol.* (2004) 186:4940–50. 10.1128/jb.186.15.4940-4950.2004 15262931 PMC451642

[59] Pugin B Barcik W Westermann P Heider A Wawrzyniak M Hellings P A wide diversity of bacteria from the human gut produces and degrades biogenic amines. *Microb Ecol Health Dis.* (2017) 28:1353881. 10.1080/16512235.2017.1353881 28959180 PMC5614385

[60] Oliphant K Allen-Vercoe E. Macronutrient metabolism by the human gut microbiome: major fermentation by-products and their impact on host health. *Microbiome.* (2019) 7:91. 10.1186/s40168-019-0704-8 31196177 PMC6567490

[61] Morishita T Tamura N Makino T Kudo S. Production of menaquinones by lactic acid bacteria. *J Dairy Sci.* (1999) 82:1897–903. 10.3168/jds.S0022-0302(99)75424-X 10509247

[62] LeBlanc JG Laiño JE del Valle MJ Vannini V van Sinderen D Taranto MP B-Group vitamin production by lactic acid bacteria – current knowledge and potential applications. *J Appl Microbiol.* (2011) 111:1297–309. 10.1111/j.1365-2672.2011.05157.x 21933312

[63] Soto-Martin EC Warnke I Farquharson FM Christodoulou M Horgan G Derrien M Vitamin biosynthesis by human gut butyrate-producing bacteria and cross-feeding in synthetic microbial communities. *mBio.* (2020) 11:e886–820. 10.1128/mBio.00886-20 32665271 PMC7360928

[64] Groschwitz KR Hogan SP. Intestinal barrier function: molecular regulation and disease pathogenesis. *J Allergy Clin Immunol.* (2009) 124:3–20; quiz 21–2. 10.1016/j.jaci.2009.05.038 19560575 PMC4266989

[65] Tannock GW Taylor C Lawley B Loach D Gould M Dunn AC Altered transcription of murine genes induced in the small bowel by administration of probiotic strain Lactobacillus rhamnosus HN001. *Appl Environ Microbiol.* (2014) 80:2851–9. 10.1128/AEM.00336-14 24584241 PMC3993288

[66] Kawasaki T Kawai T. Toll-like receptor signaling pathways. *Front Immunol.* (2014) 5:461. 10.3389/fimmu.2014.00461 25309543 PMC4174766

[67] Reinshagen M Rohm H Steinkamp M Lieb K Geerling I Von Herbay A Protective role of neurotrophins in experimental inflammation of the rat gut. *Gastroenterology.* (2000) 119:368–76. 10.1053/gast.2000.9307 10930372

[68] Nikoletopoulou V Sidiropoulou K Kallergi E Dalezios Y Tavernarakis N. Modulation of autophagy by bdnf underlies synaptic plasticity. *Cell Metab.* (2017) 26:230–42.e5. 10.1016/j.cmet.2017.06.005 28683289

[69] Matsuzawa-Ishimoto Y Hwang S Cadwell K. Autophagy and inflammation. *Ann Rev Immunol.* (2018) 36:73–101. 10.1146/annurev-immunol-042617-053253 29144836

[70] Wong M Ganapathy AS Suchanec E Laidler L Ma T Nighot P Intestinal epithelial tight junction barrier regulation by autophagy-related protein ATG6/beclin 1. *Am J Physiol Cell Physiol.* (2019) 316:C753–65. 10.1152/ajpcell.00246.2018 30892937 PMC6580157

[71] Huang L Zhang D Han W Guo C. High-mobility group box-1 inhibition stabilizes intestinal permeability through tight junctions in experimental acute necrotizing pancreatitis. *Inflam Res.* (2019) 68:677–89. 10.1007/s00011-019-01251-x 31139836

